# Medical Items and News of Interest to the Physician

**Published:** 1876-10

**Authors:** 


### Medical Items and News,

### For the Use of Physicians.

CULLED FROM THE STANDARD MEDICAL JOURNALS OF THE WORLD.

NOTE.—These formulæ are intended only for the regular practitioner of medicine, and should be employed only by him, or under his immediate supervision.

**Poisoning by Belladonna, and Cure.**

Dr. H. L. Horton, of Morrisania, N. Y., reports the case of a boy two and a half years old who swallowed 45 grains extract of belladonna, and who was cured of the effects by four teaspoonfuls of laudanum.

**Antidote to Strychnia.**

The East Indian physicians recommend nicotia as the surest antidote, which is given in exceedingly small quantities in sherry, several times a day. In default of nicotia, a decoction of tobacco leaves (½ ounce to a pint), is given.

**Toothache.**

Acid carbol. sat. sol.

Tr. opii, } āā. 2 parts.

Extr. fl. aconiti, }

Ol. menth. pip., 1 part.

Cotton moistened with this, is to be introduced into the hollow tooth.

**Rheumatic Mixture.**

℞

Sod. et Pot. Tart, ℥ ss.

Potass. Nitr., ʒ iij.

Vin. Sem. Colchici, ʒ ij.

Aquæ, q. s. ad., ℥ ij. M.

Sig., Teaspoonful at a dose.

**Solution of Salicylic Acid.**

Emlen Painter, Ph. D. (*Pacific Med. and Surg.*, April, 1876), suggests the following formula, which contains two grains of the acid to the fluid drachm: acid, salicylic., gr. xxxii; sol acet. ammon., oz. ij. Dose, a teaspoonful, to be increased or diminished as required.

**A Convenient Local Anodyne.**

Take of elastic collodion, one ounce; hydrochlorate of morphia, fifteen grains. Dissolve the morphia salt in the collodion. Spread with a camel hair brush, some of this solution on the painful part, and place some oiled silk over the spot. The effect is stated to be most satisfactory.—*London Lancet.*

**Ointment for Sycosis.**

Dr. S. Smith, of New London, Conn., advises the following formula for an ointment, which he has used for several years, with unvarying success in the cure of this intractable affection. ℞. Acid tannic, gr. xv.; sulphur, gr. xij.; aquæ, ʒ ijss. Apply a quantity the size of a pea to the affected spot every morning and night.

**Pulsatilla in Amenorrhœa.**

Dr. Pintschovius, of Ketzin, in a letter to the *Allegemeine Med. Centralzeitung*, January 30, revives pulsatilla as an emmenagogue. He has found it a "sure and convenient article for this purpose." His formula is: ℞. Extr. pulsatilla; herb. pulsat. āā q. s.; M. et div. in pill. 3 grs. each. Sig. One three times a day. It is a remedy which once enjoyed some reputation in this country.—*Med. & Surg. Reporter.*

**Hydrocele of Infants.**

Saint Germain believes that it is not advisable to subject an infant with hydrocele to even the simplest operation, until a trial has been made of a saturated solution of muriate of ammonia. Compresses dipped in such a solution should be applied. Sometimes an erythema, even slight vessication may be caused, but the part may be covered with powder, and the cure is not retarded.—*The American Practitioner.*

**Santonine as a Febrifuge.**

Dr. Francini has for some time treated intermittent fever by santonine, and regards it as a valuable substitute for quinia, preferring it to the latter in some cases. In the *Gaz. Med. Ital. Prov. Venet.*, he gives a full account of his experience. He combines it with magnesia and rhubarb, or its extract, so as to give it in powder or pills, and administers it in from three to five grains in 24 hours, divided into four doses. [Salicine, and not santonine is probably meant.]

**Lime-Water in Infantile Eczema.**

A writer in the *Bulletin de Therapeutique*, recommends lime-water in eczema of the head and impetigo of the face in children, especially chronic cases which have resisted other treatment, and states that a marked improvement is noticeable after using it for eight days. It is to be taken in quantities varying up to half a pint, according to the age of the patient, and to dust the part with carbonate of magnesia; but the latter is only necessary when the secretion is very irritant.—*Medical Brief.*

**Epilepsy.**

In the *British Med. Jour.* of July 1, W. Ainslie Hollis, M. D., refers to the criticism made by Prof. Binz,† of Bonn, to the statements made by Dr. Hollis, in 1872, regarding the value of sodic bromide in the treatment of nervous diseases. In the number of the journal referred to, Dr. Hollis reports eleven additional cases, selected from a considerable number of observations, in which the effect of treatment was to diminish the frequency of the attacks very markedly.

---

**New Reagent for Uric Acid.**

Mr. Didelot recommends, in the *Repertoire de Pharmacie*, a new and simple method for detecting uric acid in guano, bird manure, etc. A few drops of nitric acid having been poured on a small quantity of the dry substance, when a glass rod is dipped into the mixture and rubbed on a spatula or a bright piece of iron, the metal immediately assumes a Prussian blue color. The reaction, according to to the author, is extremely delicate—more so, perhaps, than in the usual ammonia test.

---

**Action of Strychnia on the Eye.**

Dr. Hippel, says that he has found by personal experience that, when given in doses of two to four milligrammes, strychnia produces the following effects, viz:—1. Increased peripheric sensibility for blue. 2. Temporary increase of visual power. 3. More distinct perception of peripheric points. 4. Lasting enlargment of the field of vision. He believes that the effect on the optic nerve is the same as that contributed to continuous electric currents on other nerves.

---

**Anæsthesia of Drunkeness Recommended.**

Professor Lynx, of Terre Haute, Indiana, announces the wonderful discovery that the best of all anæsthetics is alcohol, drank to the extent of producing coma. Several cases are described in illustration. One was that of a lady who had a "tumor on her side." She was prepared for its removal by taking two ounces of brandy every half hour till four ounces had been imbibed. Half an hour after the last dose he found her sleeping and quite unconscious, and proceeded to remove the tumor. The operation lasted fifteen minutes, during which she moaned and talked unintelligibly. She vomited once, as she had done once or twice before his arrival. The next day she was sitting up, feeling quite well. He is so delighted with his discovery that he predicts the universal adoption of it within ten years.—*Cincin. Lancet and Observer.*

**Eucalyptus Globulus in Pulmonary Gangrene.**

Of all the drugs employed by Dr. Bucquoy, (of Cochin Hospital, Paris), in pulmonary gangrene, none, he asserts, have given him better results than Eucalyptus globulus. He uses it in the form of an alcoholate—two grammes—(half a drachm), daily in a mixture of water, gum, orange-flower water, and syrup. Out of the various cases he has had under his care at Cochin, five ended in cure, whilst in all the others there was a favorable modification of the odor of the breath, the sputa, and the violent cough, after carbolic acid had failed.—*Lancet.*

---

**Ulcerated Variolous Pustules.**

Dr. Noel Gueneau de Mussy recommends to apply the following:

℞.

Acidi tannici,
Zinci oxid, āā gr xx.
Hydr. chlor. mit, gr ii.
Extr. opii, gr i.
Cerati, gr. ccc.

Between the applications the parts are to be washed with water containing a few drops of the tincture of benzoin.

---

**Pityriasis.**

Dr. Martineau asserts that a solution of forty grains of chloral to an ounce of water, applied to the scalp each morning by means of a sponge, using slight friction, and allowing it to dry, is very efficacions in pityriasis. If the disease is recent, and the lotion is uninterruptedly used for a month, he predicts a certain cure. In the chronic and more obstinate cases, he recommends the continuance of the application of the solution until the disease disappears, as its daily use produces no inconvenience, whilst it relieves the itching.—*Med. and Surg. Reporter.*

---

**Ammonia Treatment of Snake-bites.**

Dr. Halford's remedy for the treatment of snake-bites by the intravenous injection of ammonia has been frequently tried during the past year in Australia, and is reported to have been most successful. The reports from that colony state that, whatever may have been the experience of India, Australian snakes certainly do not inject enough poison to prove fatal if the ammonia treatment be resorted to in time; and it is suggested that the verdict of the Indian Commission appointed to examine and report upon Dr. Halford's method proved nothing as regards its value in dealing with the bites of the Australian snake.

**Cold in the Head.**

Dr. Ferrier, being dissatisfied with the usual formula for treating a cold in the head, namely, "sudorificcs and lying in bed," has made a trial of various substances, to be used in the form of snuffs at the beginning of an attack of nasal catarrh. He finds the following prescription very efficacious:

℞

Muriate of Morphia, gr., ij.

Acacia powder, ʒ ij.

Subnitrate of bismuth, ʒ vj.

Of the powder, one-quarter to one-half may be taken as snuff in the twenty-four hours.

---

**The Rational Treatment of Tapeworm.**

Dr. Brunton, in the *Lancet* of Dec. 4th, 1875, states that the chief points to be observed in the treatment are: A preliminary starvation of twenty-four hours, and the administration of a combination of kameela and male fern—namely, two drachms of kameela to be rubbed up with a little gum and water till the emulsion is formed, and then two drachms of oil of male fern to be added, and the whole triturated in a mortar, with a gradual addition of water till a three-ounce mixture is formed, of which half is to be given at bed-time, and the remainder four hours later. This he had never known to fail. He insists on the quality of the drugs being good.

---

**Rules in Administering Arsenic.**

In the *Medical Press and Circular*, Dr. H. Griffith gives the following rules for administering of arsenic.

1. It should never be given where there is a feverish state of the system; a quick pulse and a hot skin contra-indicate its employment.

2. It should be given shortly after meals—never on an empty stomach.

3. It should not be given in the solid form, nor should it be given in increasing doses. As a rule, five minims of Fowler's solution should be the maximum dose for an adult.

4. The dose should be diminished, or the administration altogether ceased, on the occurrence of pain in the epigastrium, nausea or irritation of the eyelids.

---

**Corpulence.**

Dr. Lucca, resident physician at the Marienbad, Bohemia, claims to have had "astonishing" results in the treatment of corpulence, by mixing two or three pounds of common carbonate of soda with a bath of Marienbad water, at a temperature of 60° or 70° Fah., one to be taken daily.

The waters of Marienbad hold in solution sulphate of soda (the chief ingredient) chloride of sodium, carbonate of soda, carbonate of magnesia, with small quantities of lime, lithia, iron, and some carbonic acid gas. They are chiefly drank, but there is bathing besides. At Marienbad, *mud* baths are in vogue, made with the water and a black substance obtained from a peat bed in the neighborhood. They stimulate the skin and disperse glandular swellings. There are also gas baths, consisting of carbonic acid gas and a little sulphuretted hydrogen, that are said to be useful in certain neuralgias and other nervous affections.

---

**Pruritus Vulvæ.**

At the meeting of the Harveian Society of London, on the 2nd of March, Dr. Wiltshire read a paper on Pruritus Vulvæ in which, following the narration of the causes which may lead or contribute to this distressing affection, the following therapeutic measures were recommended. Being a symptom of different maladies the course to be pursued in each case will necessarily differ. "Of local applications, borax was the best; and next potent, parasiticides. Ointments disagreed, and were too heating. Glycerine did not always agree. Tar or creosote were often useful. In catamenial itching borax, did good. Irritating applications were to be avoided. In diabetic pruritus, solutions of borax and careful drying of the parts were to be adopted. In the pruritus of gout, soap was inferior to oatmeal and water. Bromide of potassium internally and externally did good. Arsenic suited some cases admirably. Bran baths were often useful. A curd formed by adding *liquor plumbi* to milk, was often very agreeable.

---

**Test for Ascertaining the Presence of Blood in Liquids or Cloth.**

For physicians and clinical instructors the following method of discovering the presence of blood may be useful, especially in the examination of urine; it combines simplicity with absolute certainty. Mix in a test-tube two cubic centimetres of tincture of guaiac with an equal volume of oil of turpentine, and then add a few drops of the urine which is to be examined. If it contains any blood, even the minutest quantity, the whole mixture at once shows a more or less intensely blue color, sometimes a deep indigo, while this coloration is produced neither by normal urine nor by urine containing albumen or pus. If you wish to ascertain whether stains in linen, wood, etc., contain blood, you proceed in this way: Dissolve five grammes guaiac in 100 cubic centimetres of absolute alcohol, and filter the solution; then mix five cubic centimetres of this solution with oil. Exposed to the light it changes after a few days to a red-brown color. Although the taste is but little altered, it is important to prevent this change of color, which always indicates a liberation of iodine. In well stopped bottles, the oil remains unaltered, but it is as well not to prepare too much in advance. The taste and color furnish good criteria for its condition.—*Pharm. I.*

---

### Modus Operandi of Yellow Fever Poison.

Dr. George M. Stenberg, Assistant Surgeon U. S. A., after considering at length the various theories in regard to the causation of yellow fever, concludes that the evidence is sufficiently convincing as to the implication of the sympathetic nervous system in yellow fever poisoning; but that we have no evidence that blood-changes occur prior to the implication of the sympathetic, which marks the out-break of the disease.

The action of the poison upon the sympathetic seems to be a paralyzing one, causing the arrest of function, and producing phenomena similar to those following division of the sympathetic in any part of the body.

Whether the blood is also primarily affected by the poison, or whether the changes in it are all secondary to arrest of the processes of nutrition, secretion and excretion, may be considered a question still *sub judice.*

The fact, however, that in cases which terminate fatally within a day or two, the blood is found to be fluid, the red corpuscles more or less disorganized, and the hæmatin to have stained the tissues of the body, makes it probable that the poison also acts directly upon the blood; but that this action is of the nature of a fermentation, there is not the least evidence.

He suggests galvanization of the sympathetic, or the use of ergot, in the treatment of yellow fever. —*New Orleans Med. and Surg. Journal.*

---

### Acute Rheumatism.

Dr. Frantzel (*Charite Annalen*, Berlin, 1876), after having tried the various methods by propylamine, large doses of alkalies, and purgatives, has found his best results from following the practice of Davies in applying blisters in the neighborhood of the affected joints. He orders strips of cantharides plaster, three fingers broad, to be applied round the limbs, two inches above the wrist, elbow, knee, and ankle-joints, and below the shoulder and hip-joints, so as to avoid blistering those portions of the body which press most directly on the bed. He afterwards gives a subcutaneous injection to procure sleep; in the morning he cuts the blisters and bathes them with warm water, and puts on warm-water compresses; in the evening these are removed, and the parts dressed with simple ointment. By that evening, or often by the morning, a remarkable improvement takes place in the condition of the joints, with diminution of swelling and pain; the fever abates, and often the temperature falls to below normal; only in those cases in which the joint is not well pronounced, do the blisters remain without influence in the height of the temperature. In cases in which four or more blisters have been applied, a certain degree of strangury or anuria is the result, and the urine contains blood-corpuscles, with hyaline and granular tube-casts, but this passes off in from one to four days, without leaving a trace. He protests against the idea that this method of treatment causes much suffering to the patient, for if half a grain of morphia be injected the pain resulting is very slight.—*London Med. Record.*

---

### Angina Pectoris.

The *London Medical Record* takes from the *Paris Medical* some remarks by Professor See, of Paris, on the treatment of angina pectoris. According to him, this affection is not a neurosis, but an ischæmia, combined with pain. The treatment should, therefore, be twofold. As the pain which, by its violence, can stop the breathing, can kill the patient in a few minutes, it is to that we must first address ourselves when called during an attack. To this end, morphia hypodermically is the best, and the administration of it in this way should be continued, at least twice in the twenty-four hours, until the attack has completely disappeared. The morphia not only acts by suppressing the pain, but it assists the circulation also, and which stops the heart from receiving sufficient blood causing it to lessen. Together with morphia injections, enemata of chloral should be given to the extent of two or three grammes.

While advising chloral, Professor See cautions against the use of chloroform, which ought not to be used, owing to its tendency to paralyze the heart. Nitrite of amyl has no action as a sedative, but its effect to produce dilatation of the vessels may render it useful. Belladonna produces no effect, and the use of anti-spasmodics in a disease of such severity is absurd. Acetate of ammonia has a certain value as an excitant of the circulation, and because it acts on respiration, but it is inferior in value to morphia, Although prejudice may sometimes render it desirable to use cupping, frictions, and heat, they are really of no service. During an attack the use of the bromides is inadmissible, because they produce contraction of the blood-vessels instead of dilating them; but, like digitalis, they may be valuable during the intervals as regulators of the circulation. Arsenic, however much vaunted, does no good. Hydrotherapy is highly dangerous, either a return of the attack or cerebral congestion being results from its employment.

---

### A Useful Mode of Administering Chloroform.

In No. 226 of the *Medical Times*, a correspondent who writes from London, recommends the following simple form of anæsthetic apparatus:

"With many practitioners it must often happen that they must administer chloroform and perform their obstetric operations single-handed. Here the administration of the anæsthetic causes great anxiety.

"Still, for obstetric purposes, chloroform is yet chiefly used, and such use has been found singularly free from danger. A pleasant, safe and economical method of administering it will be found in placing a handkerchief in an ordinary tumbler, and then dropping some chloroform upon the handkerchief, the tumbler after this being held to the nose. The advantages of such plan are these. The tumbler cannot be so applied as to entirely exclude an admixture of air along each side of the nose. Then but a small quantity of chloroform is used, for as soon as each pain is over, the tumbler can be placed bottom upwards on a plate, and the loss by evaporation reduced to a minimum. So slowly does the evaporation take place, that the medical attendant can add the chloroform required at intervals himself, and so be sure of the quantity. Further, in emergencies the patient may administer the chloroform herself, the glass falling from the hand as soon as unconsciousness creeps over her. In this respect this plan is much superior to the old one of giving the anæsthetic on a handkerchief alone, which often kept its place after the hand had lost all command over it. As a means of administering chloroform in ordinary cases, the practitioner will find this plan to possess great advantages, and to secure the patient very safely against danger as well as to be most convenient and economical. Where it is necessary to intrust the administration to a nurse while the medical attendant is engaged otherwise, by this means all risk is avoided, and occasional supervision is all that is necessary."

### Employment of Aspiration in Intestinal Obstruction.

We learn from the *Medical Times and Gazette*, (London), that in a note laid before the Academie des Sciences, M. Demarquay states that the idea had occurred to him whether the same result that is obtained in gastro-enterotomy might not be attained by a simpler procedure, which might be employed by any surgeon. When an obstacle suddenly opposes the course of the contents of the intestine, gas accumulates above the obstruction, producing tympanites, which is also accompanied by nausea and vomiting, the intestinal canal becoming paralyzed by the excessive distention. If, then, at the commencement of the affection, before any local or general peritonitis has supervened, we are able to relieve the tympanites by an artificial removal of the gas, we find the intestinal movements are sometimes re-established, and with these a disappearance of the obstacle occurs. M. Demarquay has met with three cases in which he has had recourse to this procedure with success.

He gives a summary account of the last of these cases. It occurred in a man aged twenty, who was admitted into his service on February 25th, with all the signs of intestinal obstruction, the commencement of which dated from the 23d. There were present nausea, mucous vomiting, considerable tympanites, restlessness, and suffocative paroxysms due to the thrusting up of the diaphragm. On the 26th the patient's condition was still more aggravated, and four intestinal punctures having been made—two on the right and two on the left side—by means of Potain's capillary trocar, a large quantity of gas was drawn off by aspiration. The abdomen immediately became flaccid, to the great relief of the patient, and the noise of the motion of gas in the canal, due to the re-establishment of the peristaltic action was heard. As on the 27th there was still tympanites present, displaying the form of the convolutions of the intestines under the wall of the abdomen, a large quantity of gas and liquid intestinal matters were withdrawn, by means of four additional punctures. In the course of that afternoon all symptoms had disappeared.

---

### Treatment of Diphtheria.

I take the liberty of placing before your readers the treatment which has proved in my hands not only very efficacious, but almost a complete specific. For the last twelve or thirteen years we have had unequalled opportunities of watching the disease in all its stages, from the mere fibriny whiteness of the palate and tonsils to the complete obliteration of the palatine arch by false membrane internally, and the enormously enlarged submaxillary and parotid glands externally, which sometimes suppurated; death resulting in some cases from the mechanical obstruction to respiration, and in others from pure blood-poisoning, as manifested through the nervous system; the local symptoms in the throat having to a great extent disappeared, leaving both food and air passages perfectly free and open.

When called to a case of diphtheria, I first of all clear out the bowels by a dose of calomel, followed in a few hours by a little senna or Rochelle salts. If there is much heat of the skin, as will be shown by the thermometer, a warm bath will give very great relief; and after the bowels have been freely acted upon I commence with the chlorine water. To patients from ten to twelve years and upward, I give one tablespoonful; from six to twelve, one desert-spoonful, and under six, one teaspoonful, every three hours. In all cases it should be given without any addition of water. A towel saturated with cold spring-water is to be applied to the throat and neck, the cold renewed as the towel becomes warm. The patient is to have small pieces of ice on a plate by the bedside, a piece of which is to be kept constantly in the mouth. The diet should be nutritive—beef tea, milk, eggs, and such like; but no stimulent is required, unless the prostration should be alarming. In some cases which are mild, and occur in young children, a mixture containing tincture of iron, chlorate of potash, and dilute dydrochloric acid, may suffice to cure, but in no case can the same dependence be placed on it as chlorine water.

Notwithstanding the opinions of Greenhow, Brettonneau, and others of equal celebrity, as to the efficacy of caustics and gargles as topical agents, I must humbly beg leave to differ entirely with them, and disapprove *in toto* of such proceedings. I most assuredly believe them not only to be of no value, but in many cases positively injurious. If we look upon diphtheria as a blood-poison, which it undoubtedly is, we must, if possible, find an antidote and administer it. I believe we have it in the above mixture. Can we expect to cure it by a little nitrate of silver locally applied, and giving a little tonic medicine? Here is a disease, a poison in the blood, manifesting itself and increasing so rapidly in a part the free passage through which is indispensable to life, which, if not arrested, will in a few hours place the patient beyond the reach of human aid. It is not a disease which we can allow, as some fevers, to run a definite course, merely supporting the strength and guiding it to a certain turning-point. Such would, I think, be a dangerous experiment in diphtheria. Dr. J. S. Benson, in the *Canada Lancet.*

---

**Bonwill's Method of Inducing Anæsthesia.**

At an introductory lecture delivered by Prof. Adinell Hewson, at the Pennsylvania Hospital, and reported in the *Phila. Med. Times* of March 4, by Dr. T. A. Bradford; a description was given, with illustrations, of Dr. W. G. A. Bonwill's method of diminishing or allaying sensibility by rapid respirations. We quote the following portions of the article:

"You have all of you, I have no doubt, experienced the effects of rapid and deep respirations after violent running, or of blowing hard to ignite a fire—especially the confusion of sight and bewilderment of mind. These Dr. Bonwill recognized many years ago, associated with numbness of sentient nerves, as dependent on the rapidity of the respirations. Pursuing the subject, he has brought it to practical use in his profession—that of dentistry—in which he uses it constantly to diminish the sensitiveness of dentine, and even to produce such insensibility as to allow of the extraction of a molar tooth without pain. Of the latter I have had a demonstration in my own family, which has led me to the study of the subject myself, and this with the most gratifying results. I have used it in stitching wounds, in handling over-sensitive parts, and in probings and the like."

The method was tried upon two boys, who, on account of emotional excitement, failed to carry out the instructions in regard to breathing; and the writer of the report then volunteered to try the experiment before the class.

"It was his first attempt, and was made sitting erect, with his right hand resting upon a table. Breathing rapidly, (for about three minutes), was attended first with a tingling sensation of the surface, especially of the fingers, and a feeling as though the surface was all swelling. Then there followed a dizziness or confusion in the head, with consciousness well preserved, but with a feeling of inability to resist or act in an independent way. He remembered well being frequently asked by the doctor if he was hurting him, but had no recollection afterwards of the pin sticking him, much less of its having been firmly imbedded in his flesh, as he found it when he had ceased the rapid respirations and the anæsthetic effect had passed off.

"Some cases of interest, as showing important points in diagnosis, were after this brought before the class, and the rest of the hour was occupied in their discussion. After dismissing the class, Dr. Hewson went with the writer to the receiving ward, where they found waiting a boy who had fallen upon the ice an hour previously, and had sustained a severe injury to his left wrist. He was evidently suffering, and in great dread of being hurt, and the doctor directed him at once to try the rapid respirations. This, in two minutes and a half by the watch, showed some dizziness in the boy's head, when the doctor picked up the limb and moved it about with the utmost freedom, diagnosing a bad sprain of the wrist, and the absence of fracture. When the boy was recovering, he took to crying, on account, he said, of the dizziness and confusion he had experienced. Nothing could have been more satisfactory than this case in its results. He said positively *he was not* suffering any pain, the limb having been put in the easiest position possible."

---

**Freckles.**

The spots on the skin called freckles are probably of two kinds; one, occurring in persons with light complexion, from exposure to the sun, is caused by a deposit of pigment or melanin in the rete Malpighii, and is of the nature of chloasma, (or Moth), melasma, the areola of the breasts in pregnancy, etc.; while the other variety is more deeply seated, and, like the pigment of the colored races, dark moles, etc., is deposited in the corium. The former variety is comparatively transient, and is said to be as successfully treated by spirituous lotions and weak mineral acids, applied several times during the day, as by any other method. At one time and another, however, a large number of cosmetics have been recommended, of which the following represent some of the more recent:

℞

Zinci sulphocarbol, 2 parts.
Glycerine, 25 parts.
Aq. Rosæ, 25 parts.
Spiritus vini rect., 5 parts.

Dissolve and mix. The freckled skin is to be annointed with this twice daily—the ointment being allowed to stay on from one-half to one hour, and then washed off with cold water. Anæmic persons should also take a mild ferruginous tonic. In the sunlight a dark veil should be worn.

Another formula containing the sulphocarbolate of zinc is quoted from the *Bulletin Gen. de Therap.*, as follows:

A solution of corrosive sublimate either pure or mixed with cyanide of mercury is commonly employed for the removal of freckles; but a collodion containing ten per cent. of its weight of sulphocarbolate of zinc has given excellent results without being accompanied by any of the dangers attending the use of the mercurial solution.

The following formula is an excellent one:

℞

Sulphocarbolate of zinc, 1 part.
Collodion, 45 parts.
Oil of lemon, 1 part.
Absolute alcohol, 5 parts.

The sulphocarbolate of zinc should be reduced to an extremely fine powder, and should then be thoroughly incorporated with the fluid mixture.

℞

Pulv. sinapis alb., ℥ iij.
Olei amygdal, ʒ ss.
Succi limonum, enough to make a thick paste.
Mix. To be applied as an ointment.

℞

Hydrarg. perchlor., gr. v.
Acid hydrochlor., gtt. xxx.
Sacch. alb., ℥ i.
Spt. vin. rect., ℥ ij.
Aquæ rosæ, ℥ vii.

To be used as a lotion.

It is also stated that powdered nitre, moistened with water, applied to the face night and morning, will soon remove all traces of freckles.

Our grandmother used to have a remedy in buttermilk, with which, in our youthful days, our faces used to be scrubbed on Saturday nights, to clear them of sunburn and freckles for Sunday morning.—*Druggists' Circular.*

---

**Cure of Red Faces.**

Mr Balmanno Squire, in the *Medical Examiner*, discusses what he terms the "radical cure" of red face. According to him, it is quite easy in many of the most common kinds of redness disfiguring the face, and especially the region of the nose, not only to remove the eruption, but to abolish the conditions which render the skin liable to renewed blossom.

"The method I have to recommend," he says, "which in a large number of examples I have found invariably successful in all the conditions I have enumerated, is based on the method found to be sufficient in dealing with the large varicose veins so commonly to be met with on the legs, namely, division of those veins at right angles to their course. The method I practice consists in simply scarifying the skin with a 'cataract needle.'

"The needle I use is somewhat larger than an ordinary cataract needle, the head of it being about four times the customary size. With it I make parallel incisions over the effected area, spacing them at about one-sixteenth of an inch apart. In order to make the operation quite painless, and also for the purpose of ensuring precision in the drawing of the incisions, I employ Dr. Richardson's ether-spray apparatus for freezing the affected skin, not only with the view of rendering the skin insensible, but also with the view of avoiding any trickling of blood, which would interfere of course with precision in the draughtsmanship of the incisions. The incisions are carried, according to the essential nature of the lesion, either only half deep through the skin, or through its entire thickness. The hæmmorrhage, (when the skin has thawed), is quite easily controlled, or rather absolutely prevented, by firm pressure with the fingers, a layer of blotting- paper being interposed between the fingers and the scarified skin; the pressure should be kept up for between five and ten minutes, at the end of which time any tendency to bleeding will be found to have ceased. Any layer or clot should be then washed off with large camel-hair brush steeped in cold water, and the surface thereupon coated with glycerine applied with another large camel-hair brush. The needle should be sent to the instrument-maker to be sharpened after each use of it. With this precaution and a little address, it will be found that the linear incisions, (which, if deftly made, will, like the ideal mathematical line, have only length without thickness), have healed perfectly in between a week and a fortnight. After a month no trace whatever of them will remain, and the skin will be found to have regained completely its normal degree of pallor. One precaution needs to be added. After making the cuts, care must be taken not to stretch or drag upon the skin, (while exercising the subsequent pressure on it with the fingers), in a direction transverse to that of the incisions, because in such a case the wounds will be made slightly to gape, and thereupon the slightly gaping miniature incisions will become plugged, (by the time the pressure is removed), by correspondingly minute but irrevocably established wedge-shaped clots of blood. This might seem a small cause of lament; but it is not so, since, as a matter of fact, it becomes the occasion of numerous persistent linear scars, whereas, if deftly avoided, no trace whatever of the little operation remains when the month has elapsed, excepting only such an absolutely unqualified improvement as is at once gratifying to the practitioner and satisfactory to the patient.

### The Nitrate of Zinc in the Treatment of Venereal and other Lesions.

In July last, Cheron published some experiments which he had made in the treatment of mucous patches, (condylomata lata), by a rather novel method. It consisted in first applying nitrate of silver to the surface of the lesion, and then passing a stick of metallic zinc over the same. The results obtained were so remarkable, that Aubert undertook to repeat the experiments. He came to the conclusion that the effects obtained were brought about by the formation, first, of nitrate of zinc, which in turn underwent a partial decomposition into chloride of zinc, (the chlorine being obtained from the fluids and the tissues), with free nitric acid. The effective agent, therefore, was the chloride of zinc, aided by the minute amount of nitric acid. He then prepared some sticks of fused nitrate of zinc, and used them directly upon the lesions. The result was healing of the lesion in very much less time than was customary with the ordinary applications.

Impressed by the preceding, I prepared in December last some sticks of fused nitrate, and employed it freely as a cauterizing agent in private and hospital practice. The following results were obtained, transcribed in brief from my own notes, and those of my house-surgeons, Drs. Trask and Booth. The applications were usually made twice a week. More frequent applications, however, would in many cases have secured prompter results. The experiments were made during the months of Jannary and February, 1876.

Wm. F.—Simple ulcer of the leg, of three week's standing. First, second and third applications followed by improvement, and the nitrate discontinued.

J. C.—Pemphigus of two years' standing. After the rupture of the bullæ, open sores, from half an inch to two inches in diameter, would remain for from three to eight weeks without healing. As the eruption was frequent and plentiful, the patient was a mass of sores. These gently touched with the nitrate were all well in from one to two weeks.. When fresh bullæ appeared the epidermis was removed, and the raw surface touched once with the caustic. Under these applications they healed in from three to five days.

G. J.—Chancroid of one week's standing; five applications; well in eighteen days. Chronic bubo of long standing; ten applications; well in thirty-two days.

P. C. —Condylomata lata of anus, two months' standing; two applications; well in seven days. Patches of the mouth, of three weeks' duration; two applications; well in six days.

E. L.—Suppurating bubo, six weeks' open, $1\frac{1}{2}$ sec. in diameter; three applications in eight days produced no change; six subsequent applications cured in fourteen days later; in all twenty-two days.

J. R.—Bubo and sinus, $2\frac{1}{2}$ sec. long, open since August, 1875; five applications; cured in eighteen days.

J. B.—Bubo, five weeks open; ten applications; healed in thirty-eight days.

T. B.—Condylomata lata of anus; one application; well in three days.

J. S.—Irregular chancroid, with tendency to spread; four weeks' standing, six applications; well in twenty-one days.

M. S.—Syphilitic ulcer of mouth; one application; healed in three days.

M. S.—Chronic chancroid, one year old; ten applications; healed in thirty-one days.

M. T.—Chancroid, four weeks' standing; three applications, healed.

M. S.—Two recent indurated erosions, diagnosticated chancres, of right labium; one application, well in four days.

M. M. Recent bubo, $1\frac{1}{2}$ sec. in diameter; five applications; healed in twenty-one days.

M. S.—Chronic chancroid, large and indolent, duration unknown; four applications; well in twelve days.

L. L.—Recent chancroid, three weeks' duration; three applications; healed in eight days.

A. C.—Bubo, opened and treated with nitrate of zinc; five applications; healed in fourteen days.

A. M.—Chronic chancroid, of three years' standing, involving ostium vaginæ, fourchette and both labia majora; six applications; healed in twenty-six days.

After the nitrate of zinc applications, the sores were all dressed with an inert powder of starch and lycopodium, in order that the simple effects of the zinc might be observed. It is probable that the usual iodoform dressing would have secured even prompter results.

In the earlier experiments the nitrate of zinc employed was prepared by Mr. Charles Rice and by myself. In the later ones I used some sticks made for me by Dr. Squibb. These later proved more active, and were altogether better than the ones first used.—*Dr. Pifford, Surgeon to Charity Hospital.*

---

**Salicylic Acid.**

Can be very conveniently given in the doses of ten and fifteen grains by enveloping the powder in wafers or the "cachets de pain." It has been administered in this way in fifteen-grain doses for two or three weeks without causing any disturbance whatever, and at the same time producing very notable reduction of temperature.

SIRUP OF SALICYLIC ACID.

In giving this acid, the annexed formula for a sirup has been suggested:

℞.

Salicylic acid, ℥ ss.

Oil of sweet almonds, ℥ x.

Gum arabic, ℥ x.

Sirup of almonds. ℥ xij.

Orange-flower water, ℥ xij.

EXTERNAL USE.

Dr. Wagner recommends that a thin layer of finely powdered salicylic acid should be spread upon calico, and applied by means of a bandage to wounds.

POMADE.

Dr. Wagner gives the following formula:

Salicylic acid, 15 parts.
Alcohol, 30 parts.
Lard, 150 parts.

It is important to use the alcohol as a solvent; the direct mixture of the acid with the lard does not give the same good effects.

DENTIFRICES.

M. Paulcke, a pharmacien at Leipzig, prepares as a dentifrice a powder in which salicylic acid is incorporated; also an *elixir "dentifrice,"* from a solution of the acid aromatized with oil of wintergreen.

FOOT POWDER.

It is stated that salicylic acid removes the odor of sweat from the feet, without preventing the sweating; its action being to prevent the formation of butyric, valerianic, and other acids of the same family, which injure the feet. M. Paulcke, therefore, prepares with salicylic acid, soap, talc, and starch, a powder for the feet, which, while rendering them firm, is said to induce an agreeable softness, and removes all unpleasant smell.

MIXTURE.

The following formula is accredited to Prof. Wunderlich:

Salicylic acid, 1 gramme.
Oil of sweet almonds, 20 grammes.
Gum arabic, 10 grammes.
Sirup of almonds, 25 grammes.
Orange-flower water, 45 grammes.

A teaspoonful to be taken every hour. When children are sufficiently old to use a gargle, Dr. Fontheim says it may be so administered every hour.

